# Study protocol for a randomized controlled trial of fecal microbiota transplantation via different routes in children with moderate-to-severe autism spectrum disorder

**DOI:** 10.3389/fmicb.2026.1829532

**Published:** 2026-05-28

**Authors:** Yigui Zou, Liang Liu, Hu Chen, Zimu Luo, Zhongsheng Zhu, Ziyuan Li, Baixian Lin, Zeling Zhuang, Wenwen Li, Qinghua Yang, Xiao Yang, Haokui Zhou, Mingjing Luo, Dongling Dai

**Affiliations:** 1 International Medical Center, Endoscopy Center and Gastroenterology Department, Shenzhen Children's Hospital, Shenzhen, China; 2 Guangdong Medical University, Zhanjiang, Guangdong, China; 3 State Key Laboratory of Quantitative Synthetic Biology, Shenzhen Institute of Synthetic Biology, Shenzhen Institute of Advanced Technology, Chinese Academy of Sciences, Shenzhen, China; 4 Department of Biostatistics, School of Public Health, Cheeloo College of Medicine, Shandong University, Jinan, China; 5 Shenzhen Children's Hospital, Southern University of Science and Technology, Shenzhen, China; 6 Shenzhen Children's Hospital, The Chinese University of Hong Kong, Shenzhen, China; 7 Shenzhen Children's Hospital, Shenzhen University Health Science Center, Shenzhen, China

**Keywords:** Autism Spectrum Disorder, clinical protocol, fecal microbiota transplantation, gastrointestinal symptoms, microbiome, microbiome-gut-brain axis, microbiota dysbiosis

## Abstract

**Background:**

Fecal microbiota transplantation (FMT) shows promise for autism spectrum disorder (ASD) by modulating the gut-brain axis, but the optimal delivery route remains unknown. Our previous single-arm study suggested efficacy of nasojejunal FMT in children with moderate-to-severe ASD, yet could not exclude placebo effects or compare routes. This randomized controlled trial aims to determine the most effective and tolerable FMT administration route.

**Methods:**

This single-center, randomized, triple-blind, double-dummy, placebo-controlled, three-arm parallel-group trial will enroll 75 children (aged 3–16 years) with moderate-to-severe ASD [Childhood Autism Rating Scale, Second Edition (CARS-2) ≥36]. Participants are randomized 1:1:1 to: (1) FMT via nasojejunal tube + sham colonoscopy (FMT-NJT); (2) active FMT via colonoscopy with transendoscopic enteral tube placement (first session) + two subsequent infusions via the indwelling tube + sham nasojejunal intubation (FMT-C); (3) placebo via both routes (sham procedures). Three FMT/placebo sessions (5 mL/kg, max 100 mL) are administered over 5 days. Primary outcome is change in CARS-2 score from baseline to Week 24. Secondary outcomes include changes in Social Responsiveness Scale, Autism Behavior Checklist, Gastrointestinal Symptom Rating Scale, Short Sensory Profile, Children's Sleep Habits Questionnaire, gut metagenomic profiles (baseline, Weeks 2,6,12,24,48), and adverse events.

**Results:**

This is a study protocol; no results are available.

**Conclusions:**

This first head-to-head comparison of FMT routes in pediatric ASD will provide high-level evidence to guide treatment standardization, directly addressing the translational gap identified in our preliminary work.

## 1 Introduction

Autism Spectrum Disorder (ASD) is a complex neurodevelopmental disorder characterized by core features of persistent social communication deficits, restricted interests, and repetitive patterns of behavior. It is frequently accompanied by gastrointestinal (GI) symptoms, immune dysfunction, and metabolic dysregulation (Hirota and King, 2023; Wu et al., 2025). ASD affects approximately 1–2% of children worldwide, with a significant upward trend, making it a global public health challenge (Salari et al., 2022; Zeidan et al., 2022). The etiology of ASD is likely multifactorial, involving interactions between genetic susceptibility and environmental factors (Hirota and King, 2023; Manoli and State, 2021). Current primary interventions for ASD predominantly rely on behavioral and educational therapies, with a notable lack of pharmacological agents that effectively target core symptoms. The efficacy of intensive behavioral interventions requires further validation (Kodak and Bergmann, 2020), while existing medications are limited by adverse effects (Koch and Demontis, 2022). Consequently, there is an urgent need to explore safe and effective therapeutic strategies.

Recent advances in understanding the microbiome-gut-brain axis (MGBA) have highlighted the role of gut microbiota dysbiosis and its impact on neurodevelopment (You et al., 2024; Morton et al., 2023). The MGBA facilitates bidirectional communication between the gut and the brain via neural, immune, endocrine, and metabolic pathways. Substantial evidence indicates that intestinal dysbiosis is a key susceptibility factor in ASD (Ullah et al., 2023). Characteristic dysbiosis in ASD includes reduced microbial diversity, depletion of beneficial bacteria, and enrichment of potential pathogens (Caputi et al., 2024). This dysbiosis is thought to drive immune dysregulation and neuroinflammation, core features of ASD pathophysiology, and alter the gut metabolome, thereby modulating brain function (Ullah et al., 2024; Goncalves et al., 2024) . The compelling link between gut microbiota and ASD pathophysiology has spurred interest in microbiota-targeted therapies.

Fecal Microbiota Transplantation (FMT) has emerged as a promising approach to restore intestinal microbial balance, demonstrating potential efficacy in alleviating both core ASD symptoms and co-occurring GI disturbances in preliminary studies (Tan et al., 2021; Li et al., 2022). Animal models of ASD show that FMT from healthy donors can reduce anxiety-like and repetitive behaviors, improve social interaction, and alleviate memory deficits, correlating with positive changes in gut microbiota and brain biochemistry (Chen et al., 2020). Early human studies, including open-label trials and prospective cohorts, have reported improvements in behavioral scores, GI symptoms, and sleep disturbances following FMT, alongside shifts in gut microbial ecology (Wu et al., 2025; Kang et al., 2017; Li et al., 2024b,a; Kang et al., 2019). A recent study found that colonoscopic FMT significantly improved GI symptoms and behavioral scores (CARS) in children with ASD at 8 weeks and 6 months, with sustained benefits up to 18 months, though no significant difference was observed in the primary ADOS outcome (Varnas et al., 2025).

Despite promising results, critical gaps remain. Various FMT protocols (e.g., colonoscopic infusion, oral capsules, nasojejunal tube) have been employed, but a direct comparison of their efficacy and associated microbial engraftment profiles is lacking. The optimal delivery route for maximizing clinical outcomes and ensuring beneficial microbiota colonization in children with ASD is not established. This study aims to address this pivotal question by directly comparing two invasive FMT delivery routes—nasojejunal tube (NJT) to the jejunum and colonoscopic infusion to the cecum—against a sham-controlled placebo group in a rigorously designed trial. Our previous single-arm exploratory study established a standardized operational protocol for nasojejunal tube-delivered FMT provided preliminary evidence for the potential of NJT-delivered FMT, and preliminary findings demonstrated its potential in improving core symptoms and gastrointestinal comorbidities in children with moderate-to-severe ASD (Lin et al., 2025). However, the design of that study could not exclude placebo effects and did not compare different administration routes. The optimal delivery route for FMT and its impact on clinical efficacy and microbial engraftment patterns remain elusive.

## 2 Methods and analysis

### 2.1 Trial design and registration

#### 2.1.1 Trial design

**Figure 1:**
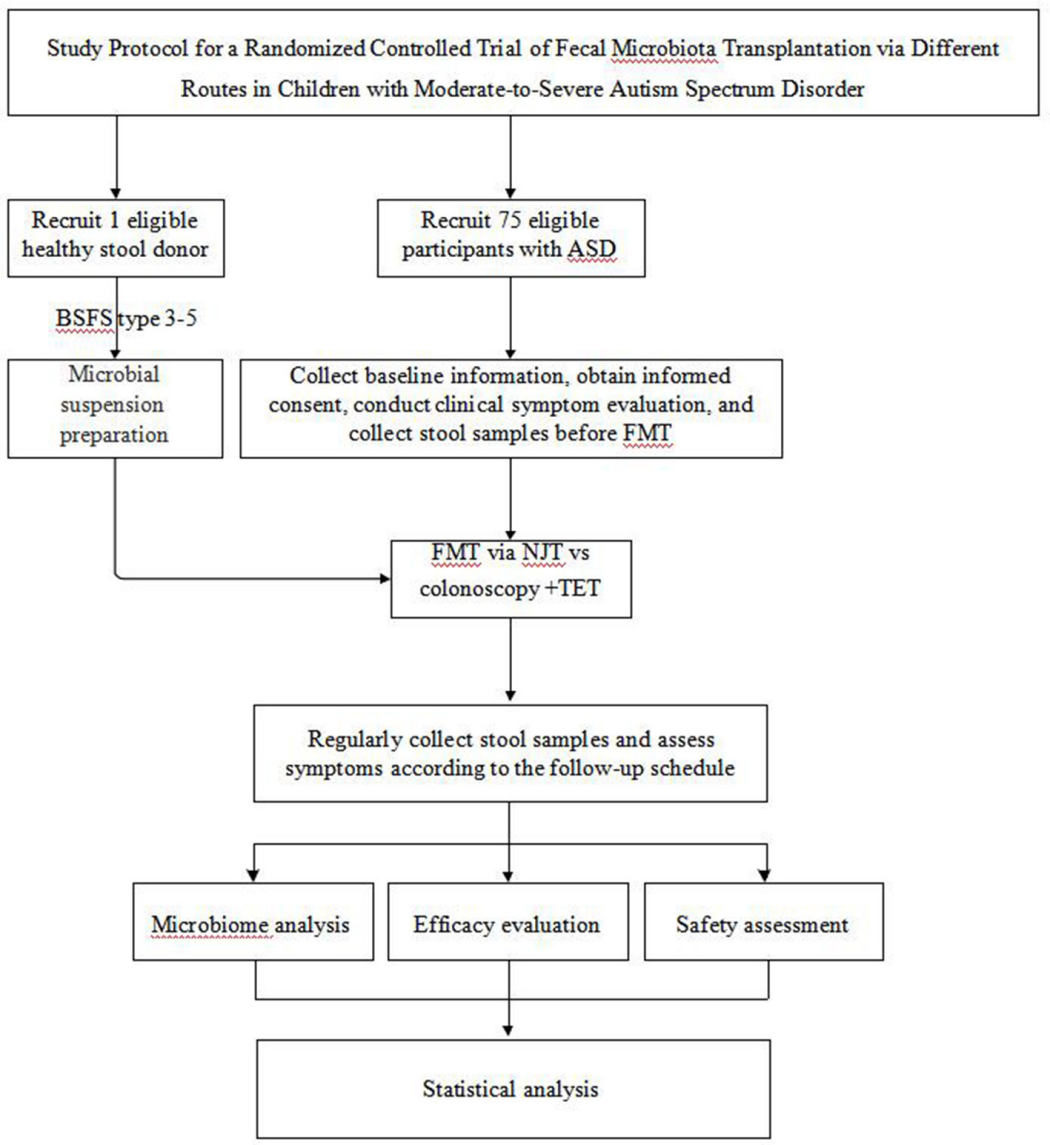
Workflow chart.

This is a single-center, prospective, randomized, double-dummy, triple-blind, placebo-controlled, three-arm, parallel-group, superiority trial. The study workflow is summarized in Figure 1.

#### 2.1.2 Trial registration

The study is registered on ClinicalTrials.gov (Identifier: NCT07381374). The protocol adheres to the principles of the Declaration of Helsinki and has been approved by the Institutional Review Board of Shenzhen Children's Hospital (Approval No.: 202500102). Written informed consent will be obtained from all participants' legal guardians.

### 2.2 Objectives

#### 2.2.1 Primary objective

To compare the efficacy of FMT delivered via NJT vs. colonoscopy, both as add-on therapies to existing behavioral intervention, against a placebo control, in improving social interaction and communication (as measured by the change in the total score of the CARS-2) in children with moderate-to-severe ASD from baseline to Week 24.

#### 2.2.2 Secondary objectives

(1) To assess the effects of both FMT routes on overall social responsiveness (SRS), aberrant behaviors (ABC), sensory processing (SSP), emotion regulation (ERC), and sleep quality (CSHQ).
(2) To evaluate the improvement of GI symptoms (GSRS) following FMT.
(3) To investigate gut microbiota engraftment dynamics and their correlation with clinical efficacy using metagenomic sequencing.
(4) To evaluate the safety and tolerability of both FMT routes in children with ASD.

### 2.3 Participants

#### 2.3.1 Inclusion criteria

Participants must meet all of the following criteria:

(1) Aged 3–16 years, any gender.
(2) Meets DSM-5 diagnostic criteria for moderate-to-severe ASD. With a CARS-2 (Schopler et al., 2010) total score ≥36, confirmed by the Autism Diagnostic Observation Schedule, Second Edition (ADOS-2) (Lord et al., 2012). The module 1–4 selection of ADOS-2 is strictly standardized according to the age and language ability of the subjects.

DSM-5 Diagnostic Criteria for ASD:

(a) Onset in early developmental stages.
(b) Persistent deficits in social communication and interaction across multiple contexts, accompanied by restricted, repetitive patterns of behavior, interests, or activities.
(c) Symptoms cause clinically significant impairment in social, occupational, or other critical functional areas.
(d) Symptoms are not better explained by intellectual disability or global developmental delay.

CARS-2 Scoring System:

(a) Total score < 30: non-autistic.
(b) Total score 30–36: mild-to-moderate autism.
(c) Total score ≥36: moderate-to-severe autism.

(3) Has been receiving and plans to maintain a stable behavioral intervention regimen (≥3 months prior to enrollment, with no major changes planned for the next 6 months).
(4) Legal guardians fully comprehend the trial's informed consent and voluntarily provide written informed consent.
(5) Compliance with follow-up visits, examinations, and specimen collection.
(6) No use of probiotic/prebiotic/synbiotic supplements within 3 months prior to enrollment.

#### 2.3.2 Exclusion criteria

Participants will be excluded if any of the following criteria apply:

(1) Antibiotic usage within 1 month prior to enrollment.
(2) Presence of fever (axillary temperature ≥37.5 °C).
(3) Dependency on tube feeding.
(4) Diagnosis of severe GI conditions requiring immediate intervention (e.g., life-threatening intestinal obstruction, perforation, hemorrhage, ulcerative colitis, Crohn's disease, celiac disease, or eosinophilic esophagitis).
(5) Diagnosis of severe malnutrition, underweight status (BMI-for-age < 3rd percentile).
(6) History of severe allergic reactions or known allergy to FMT preparation components.
(7) Monogenic disorders (e.g., Fragile X syndrome, Rett syndrome).
(8) Comorbid psychiatric diagnoses as a primary diagnosis, including depression, developmental speech/language disorders, intellectual disability, attention-deficit/hyperactivity disorder (ADHD), selective mutism, reactive attachment disorder, or childhood schizophrenia.
(9) Presence of severe systemic organic diseases, immunodeficiency disorders, or chronic infections.
(10) Contraindications to NJT placement or endoscopy.
(11) Unlikely to complete the study follow-up.

#### 2.3.3 Withdrawal criteria

Participants will be withdrawn from the study if:

(1) The legal guardian or participant withdraws informed consent due to inability or unwillingness to continue the trial.
(2) The participant fails to complete scheduled follow-up assessments.
(3) Clinical deterioration renders continued participation unsafe or impractical.
(4) Intolerable adverse reactions occur, and the investigator deems the risks of continued participation to outweigh potential benefits.

Note: Participants may withdraw from the trial at any time without justification. Investigators will document the reasons for withdrawal (if provided) in the case report form and complete all feasible assessments. Participants experiencing adverse reactions will receive appropriate medical management based on their condition.

#### 2.3.4 Elimination criteria

Participants will be eliminated from the final analysis if:

(1) No data are recorded throughout the trial period.
(2) Serious protocol deviations occur.
(3) Medications or interventions that could confound trial results are administered during the study.

### 2.4 Sample size and randomization

#### 2.4.1 Sample size calculation

The sample size for this prospective, randomized, three-arm study was determined based on the primary outcome of the change in the CARS-2 total score from baseline to 24 weeks post-intervention. The calculation is primarily informed by data from the open-label study by Kang et al. (2017) on FMT for children with ASD.

In that study, FMT led to a mean improvement in CARS-2 scores. To define a clinically meaningful difference for this randomized controlled trial, we estimated that the active FMT intervention would yield a 30% greater improvement compared to the placebo control. Based on a typical baseline CARS-2 score of 34.5, this translates to a target between-group difference (δ) of 2.5 points to be detected.

The standard deviation (σ) for the change score is estimated to be 3.5 points, based on the variability observed in prior studies. For the planned Analysis of Covariance (ANCOVA) which uses baseline score as a covariate, the correlation between baseline and follow-up measurements (ρ) is assumed to be 0.5, a conventional estimate for such scales.

Using a one-way ANCOVA model for three groups, with a significance level (α) of 0.05 (two-sided) and a desired statistical power (1-β) of 80%, the required sample size per group (*n*) was calculated using the formula appropriate for ANCOVA: *n* = $\frac{{2\times \left(Z_{1-\alpha 2}+Z_{1-\beta }\right)}^{2}{\times \sigma }^{2}\times \left(1-\rho^{2}\right)}{\delta^{2}}$, Where Zα/2 = 1.96 and Zβ = 0.84. This calculation yielded an initial requirement of 21 participants per group.

To account for a potential dropout rate of approximately 15% during the 24-week study period, the sample size was adjusted:

Adjusted sample size per group = *n* / (1 - dropout rate) = 21 / (1 - 0.15) ≈ 24.7

Therefore, we plan to enroll 25 participants per group. With three groups, the total target sample size is 75 participants. This enrollment provides sufficient statistical power to evaluate the primary outcome while accounting for anticipated attrition.

#### 2.4.2 Randomization

Eligible participants will be randomized in a 1:1:1 ratio to the three groups using a central computerized block randomization system (with randomly varying block sizes). Randomization will be stratified by age group (3–6 years, 7–12 years, 13–16 years) and baseline CARS-2 severity classification (Moderate, Severe) to ensure balance across groups.

### 2.5 Study procedures

#### 2.5.1 Recruitment and screening of a healthy fecal donor

A single healthy donor of 3–16 years will be rigorously screened to ensure safety and consistency. Screening includes:

a) Comprehensive screening includes periodic questionnaires, medical history review, and physical examinations to exclude infectious diseases, metabolic syndrome, GI disorders, and neurological conditions.
b) Serological tests to rule out HIV-1/2, hepatitis A/B/C, and syphilis.
c) Stool tests for bacterial pathogens (C.difficile toxin, Campylobacter, Salmonella, toxigenic E. coli, Vibrio, Yersinia, Listeria, methicillin-resistant Staphylococcus aureus, vancomycin-resistant Enterococcus), parasites (Giardia, Cryptosporidium, Cyclospora, Isospora), and viruses (rotavirus A, adenovirus, norovirus).
d) Assessment of metabolic health via physical exams and serological tests (fasting glucose, lipid profile, liver function tests, high-sensitivity C-reactive protein).
e) Assessment of autoimmune risks via fluorescent antinuclear antibody test.

^*^Note: Detection methods for the pathogens: stool culture for aerobic and anaerobic bacteria (Salmonella, Shigella, Campylobacter, Yersinia, Vibrio, Listeria, toxigenic E. coli); chromogenic culture for MRSA and VRE; PCR for C. difficile toxin genes, rotavirus, adenovirus, norovirus, and for specific bacterial virulence factors; microscopy and antigen detection (EIA) for parasites (Giardia, Cryptosporidium, Cyclospora, Isospora); and 13^c^ breath test for H. pylori.

Before each donation, donors complete questionnaires addressing dietary habits, lifestyle, bowel habits, and medication use. Full pathogen screening will be repeated quarterly. Qualified donors undergo metagenomic sequencing of fecal samples to monitor microbial composition and abundance quarterly. Batches with >20% change in dominant phyla relative abundance will be excluded. In our preliminary studies, we have identified three super-donors. Therefore, this study will primarily use fecal microbiota suspension from a single donor for FMT to ensure homogeneity of the study. If this donor becomes unable to provide fecal samples due to illness or other reasons, alternative donors will be considered as replacements. The new donor will undergo identical screening and qualification. Participants who receive FMT from different donors will be analyzed separately in a sensitivity analysis to assess potential donor effects.

#### 2.5.2 . Recruitment of ASD children

A total of 75 children aged 3–16 years with moderate-to-severe ASD (CARS-2 score ≥36) will be recruited. Diagnosis will be confirmed by a qualified clinician using DSM-5 criteria and the CARS-2. After obtaining written informed consent from guardians, eligible participants will be followed up routinely at the psychology department and complete baseline assessments within 2 weeks prior to FMT. Subsequently, they were randomly allocated into 3 groups based on the order of their enrollment, with 25 participants in each group. The 3 groups will be comparable with no significant differences in age or gender.

### 2.6 Interventions

The study involves add-on FMT or placebo procedures. To minimize known confounding factors that can influence the gut microbiome, all participants continue their pre-existing stable behavioral interventions regimen and avoid introducing major dietary changes throughout the study. A baseline 3-day dietary log will be collected. All concomitant medications and the use of supplements will be recorded at each study visit. While household environment cannot be controlled, data on significant dietary patterns and concomitant medications will be collected and considered as potential covariates in exploratory statistical models.

#### 2.6.1 Donor fecal collection

Fresh donor stool is collected and immediately transported in anaerobic bags to the FMT processing laboratory.

Fresh stool is visually assessed using the Bristol Stool Form Scale as shown in Table 1 (BSFS, type 3–5 are acceptable; type 1, 2, 6, or 7 result in sample rejection). If transportation compromises visual assessment, donor-provided Bristol scores are used.

**Table 1:**
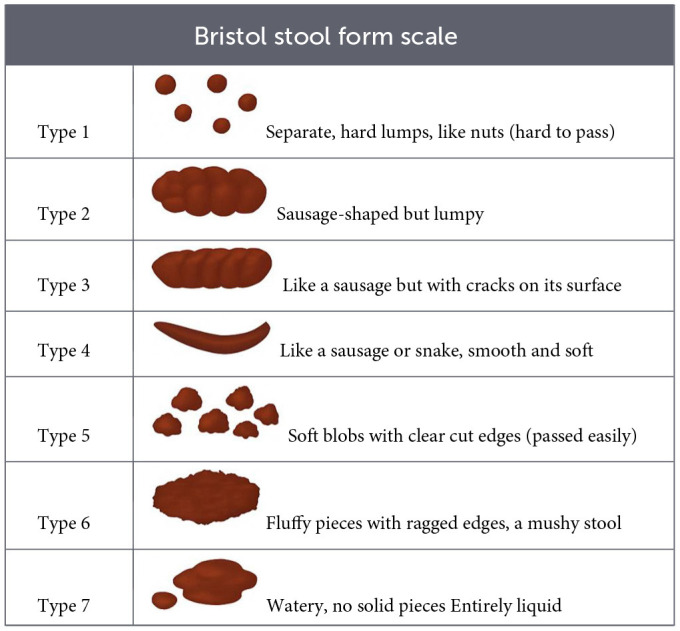
Bristol stool form scale.

#### 2.6.2 Donor fecal preparation

FMT preparation is conducted in a dedicated laboratory at Shenzhen Children's Hospital.

##### 2.6.2.1 Active FMT preparation

Donor stool is homogenized with saline and 10% glycerol. It is then filtered, aliquoted into and stored at −80 °C.

**Step 1**: Stool is weighed, mixed with 0.9% sterile saline at a 1:3 ratio and homogenized for 5 min under anaerobic conditions. Adjustments are made for samples < 100 g.

**Step 2**: The homogenate is filtered through a membrane-equipped bag using a stomacher (2 min) to remove particulates.

**Step 3**: Filtrate is transferred to sterile cryovials, and 80% glycerol is added to a final concentration of 10%.

**Step 4**: Cryovials are labeled with donor ID and preparation time.

**Step 5**: Preparations are stored at −80 °C (stable for ≤ 6 months) or −20 °C (use within 1–4 weeks). It is thawed at room temperature (< 6 h) or in a water bath ( ≤ 37 °C) before use.

**Step 6**: A “Donor Sample Processing Record” is completed.

**Residual Sample Handling**: Remaining filtrate is aliquoted into 2 mL/15 mL centrifuge tubes with 10% glycerol and stored at −80 °C for quality control and post-treatment analysis. 1 mL aliquots of saline and glycerol from the same batch are archived in 2.0 mL tubes.

##### 2.6.2.2 Placebo preparation

We use sterile 0.9% saline supplemented with food-grade coloring and flavoring agents (e.g., cocoa powder and vanillin) to exactly match the active FMT preparation.

#### 2.6.4 Pre-FMT preparation

Patients initiated a colorless and residual-free semi-liquid diet 3 days prior to FMT and bowel preparation with oral polyethylene glycol 4,000 powder 1 day prior to FMT. Bowel preparation adequacy was confirmed when colorless or light yellow transparent watery stools were discharged.

Omeprazole is administered intravenously before the procedure to suppress gastric acid and maximize the viability of the transplanted microbiota during nasojejunal passage.

#### 2.6.5 Group allocation and procedure (double-dummy design)

##### 2.6.5.1 Group 1 (FMT-NJT)

###### 2.6.5.1.1 Active procedure

Under endoscopic guidance, a NJT is placed with the tip positioned past the Ligament of Treitz. Active FMT suspension (5 mL/kg, max 100 mL) is infused slowly.

###### 2.6.5.1.2 Sham procedure

Under anesthesia, a sham colonoscopy is performed. The colonoscope is inserted to the rectosigmoid junction (approx. 15–20 cm), with sterile saline (approx. 20 mL) insufflation to simulate mucosal inspection. The dwell time matches the real colonoscopy group. No FMT is infused.

##### 2.6.5.2 Group 2 (FMT-C)

###### 2.6.5.2.1 Sham procedure

A sham nasojejunal intubation is performed. The tube is placed into the stomach (approx. 55–60 cm) and secured to the face, but not advanced into the duodenum. Placebo preparation (sterile saline, 5 mL/kg, max 100 mL) is infused.

###### 2.6.5.2.2 Active procedure

Under anesthesia, a full colonoscopy is performed to the terminal ileum. During this first session, active FMT preparation (5 mL/kg, no more than 100 ml) is infused into the cecum via the biopsy channel. A transendoscopic enteral tube (TET) is then placed with its tip positioned in the cecum and securely fixed using endoscopic clips. For the second and third treatment sessions (Days 2 and 4), the active FMT preparation is infused through this indwelling TET without the need for repeat colonoscopy or anesthesia.

##### 2.6.5.3 Group 3 (control)

###### 2.6.5.3.1 Sham procedure 1

Identical to Group2′s sham nasojejunal intubation, with infusion of placebo.

###### 2.6.5.3.2 Sham procedure 2

Identical to Group1′s sham colonoscopy, with no FMT infusion.

###### 2.6.5.3.3 Treatment schedule

FMT is repeated 3 times over 5 days (once every other day). For Group 2 (FMT-C), the first infusion is performed under colonoscopic guidance with TET placement. The subsequent two infusions are administered via the indwelling TET.

#### 2.6.6 Blinding (triple-blind)

This study is designed to achieve blinding of the participants and their guardians, outcome assessors, and data analysts to group allocation.

Participants and Guardians: All interventional procedures (both active and sham) are performed under sedation/anesthesia. The use of a matched placebo, identical in appearance and smell to the active FMT preparation, minimizes the potential for participants to perceive differences based on the treatment experience. Participants and their guardians are informed that they are receiving an “intestinal microbiota modulation therapy” but remain unaware of the specific administration route and whether they are receiving the active treatment or placebo.

Outcome Assessors: The clinicians and psychologists administering the primary (CARS-2) and secondary behavioral outcome scales are fully blinded to group assignment. They are not involved in the FMT administration process and have no access to procedural notes or reports that might reveal the assigned intervention.

Data Analysts: Statisticians remain blinded until the final database is locked for the primary analysis.

Intervention Team: An “Intervention Team” of endoscopists and nurses performs all intubation, endoscopic, and infusion procedures. As the nature of the procedure itself cannot be concealed from the operators, this team is aware of the assigned procedural route. However, to minimize bias, the following measures are implemented:

All substances for infusion (active FMT or placebo) are prepared, aliquoted, and visually standardized by an independent pharmacy unit according to the randomization code.

The Intervention Team is instructed only to administer the liquid corresponding to a specific code and remains blinded to whether it is the active preparation or placebo.

Members of the Intervention Team are strictly prohibited from discussing any procedural details with participants, guardians, or outcome assessors.

#### 2.6.7 Concomitant care and lifestyle

Guardians are instructed to maintain the child's typical diet and avoid initiating major dietary changes during the study. A 3-day dietary log is collected at baseline. All concomitant medications and supplements are recorded at each visit.

#### 2.6.8 Study flow and assessments

The schedule of assessments is detailed in Table 2 (represented textually below).

**Table 2:** The schedule of assessments.

Activity/ assessment	Screening	Baseline (day 0)	Intervention^*^	Week 2	Week 6	Week 12	Week 24	Week 48
Informed consent	X							
Eligibility criteria	X							
Demographics/history	X							
Physical exam/vitals	X	X	X	X	X	X	X	X
Randomization			X					
Lab tests^†^		X			X		X	X
FMT/placebo procedures			X					
ADOS-2	X							
CARS-2	X						X	X
SRS-2, ABC, SSP, CSHQ	X					X	X	X
GSRS, DSR	X	X	Daily	X	X	X	X	X
Recipient fecal collection	X	X	X	X	X	X	X	X
Stool for metagenomics	X			X	X	X	X	X
AEs^‡^			X	X	X	X	X	X

^*^Intervention: during FMT, D0–D4.

^†^Lab tests include routine blood, urine, and stool test, liver function (ALT, AST), renal function (BUN, Creatinine), coagulation profile, and ECG (baseline). Follow-up labs at week 6, 24 and 48 include inflammatory markers (Complete blood count (CBC), hs-CRP, ESR) and metabolic panels (Fasting glucose, HbAlc, Fasting lipid profile, liver function. Renal function).

^‡^Long term safety and efficacy including immune, metabolic, and neurodevelopmental parameters.

ABC, Aberant Behavior Checklist; CARS-2, Childhood Autism Rating Scale 2; DSR, Daily Stool Record; FMT, Fecal Microbiota Transplantation; GSRS, Gastrointestinal symptom Rating Scale; SRS, Social Responsiveness Scale.

Screening/Baseline (Week−4 to Day 0): Informed consent, eligibility screening, medical history, demographics, physical exam, vital signs, laboratory tests (CBC, chemistry, coagulation, ECG), collection of all baseline scales (ADOS-2, CARS-2, SRS, ABC, GSRS, SSP, CSHQ), and baseline stool sample for metagenomics. Chemistry refers to serum biochemistry panel including liver function (ALT, AST), renal function (BUN, creatinine), fasting glucose, and electrolytes. Coagulation refers to prothrombin time (PT), activated partial thromboplastin time (APTT), and international normalized ratio (INR).

Intervention Period (Week 0): Hospitalization. Administration of FMT/placebo procedures (three sessions). Daily recording of adverse events (AEs), GI symptoms (GSRS, Daily Stool Record - DSR), and vital signs.

##### 2.6.8.1 Follow-up Visits

Weeks 2, 6, 12: Efficacy and safety assessments, AEs assessments, vital signs, GSRS, DSR, stool sample metagenomics (CARS-2 at Week 12).

Weeks 24 (Primary Endpoint): Efficacy and safety assessments, AEs assessments, vital signs, laboratory tests (microbial dynamics, immune/metabolic panels), GSRS, DSR, stool sample, CARS-2, SRS, ABC, SSP, CSHQ.

Weeks 48 (Long-term): Efficacy and safety assessments, AEs assessments, vital signs, laboratory tests (microbial dynamics, immune/metabolic panels), GSRS, DSR, stool sample, CARS-2, SRS, ABC, SSP, CSHQ.

#### 2.6.9 Adverse event management plan

All AEs will be graded for severity according to the Common Terminology Criteria for Adverse Events (CTCAE) v5.0 (Grades 1–5: Mild, Moderate, Severe, Life-threatening, Death).

##### 2.6.9.1 Expected AEs

Procedure-related: Sedation risks, sore throat, epistaxis, abdominal bloating/pain, GI bleeding or perforation (very rare).

FMT-related: Diarrhea, constipation, bloating, belching, fever, nausea, vomiting. These are typically self-limiting (Grade 1–2).

##### 2.6.9.2 Management protocol

All AEs will be recorded in detail, including time of onset, severity, duration, relationship to intervention, and outcome. For Grade 1–2 AEs, management will primarily involve observation and symptomatic support. Any Grade 3 or higher SAE, or any unexpected serious event potentially related to FMT (e.g., opportunistic infection, bacteremia), must be reported to the Principal Investigator and the Ethics Committee within 24 h. The event will be investigated immediately, and unblinding may be performed if necessary to guide clinical management. The study will be temporarily halted for immediate safety review if a serious infectious event unequivocally related to FMT occurs.

### 2.7 Outcome measures

#### 2.7.1 Primary outcome

CARS-2 Total Score Change: The change from baseline to Week 24 in the total score of the CARS-2, administered by blinded, trained assessors.

#### 2.7.2 Secondary outcomes

(1) Behavioral and Symptom Scales: Change from baseline in scores on:
  - Social Responsiveness Scale, 2nd Edition (SRS-2) (Cen et al., 2017), the Chinese revised version.
  - Autism Behavior Checklist (ABC) (Kat et al., 2020), the Chinese revised version.
  - Childhood Autism Rating Scale 2 (CARS-2).
  - Short Sensory Profile (SSP) (Li and Wang, 2015), the Chinese revised version.
  - Children's Sleep Habits Questionnaire (CSHQ) (Li et al., 2007), the Chinese revised version.
(2) Gastrointestinal Symptoms: Change from baseline in:
  - Gastrointestinal Symptom Rating Scale (GSRS) (Qin et al., 2023) score, the Chinese revised version.
  - Daily Stool Record (DSR) parameters (e.g., % days with abnormal bowel movements).
(3) Microbiome Analysis: Changes in gut microbiota composition (alpha/beta diversity, specific taxon abundances) and functional potential from baseline to follow-up time points (W2, W6, W12, W24, W48) via shotgun metagenomic sequencing (target depth ≥10 Gbp/sample). Bioinformatic analysis will include taxonomic profiling (Kraken2/MetaPhlAn), functional profiling (HUMAnN 3.0 against KEGG), and correlation with clinical changes.
(4) Safety: Incidence, severity (graded by CTCAE v5.0), and relationship to intervention of all adverse events (AEs) and serious adverse events (SAEs).

### 2.8 Data management and statistical analysis

#### 2.8.1 Data management

Data will be collected using electronic case report forms (eCRFs) in a secure, compliant electronic data capture (EDC) system. Double data entry or equivalent quality control procedures will be employed. The database will be locked before the blinding code is broken for analysis.

#### 2.8.2 Analysis sets:

Full Analysis Set (FAS): Will include all randomized participants, analyzed according to the intention-to-treat (ITT) principle.

Per-Protocol Set (PPS): Will include all participants who complete the intervention and the primary endpoint assessment without major protocol deviations.

Safety Set (SS): Will include all participants who receive at least one intervention procedure.

#### 2.8.3 Statistical methods

Primary Endpoint Analysis: Analysis of Covariance (ANCOVA) will be used to compare the change in CARS-2 scores at Week 24 across the three groups, with baseline scores as a covariate. If the overall ANCOVA test is significant, pairwise comparisons (e.g., FMT-NJT vs. Control, FMT-C vs. Control) will be conducted with adjustment for multiple comparisons (e.g., Bonferroni correction).

Secondary Endpoints Analysis: Continuous variables will be analyzed using ANCOVA or mixed-effects models for repeated measures. Categorical variables will be analyzed using Chi-square or Fisher's exact tests.

Microbiome Data: Differential abundance analysis (e.g., LEfSe, DESeq2) and correlation analyses (Spearman's rank) will be used to link microbial features with clinical outcomes.

Safety Analysis: Statistical analyses will be performed using SPSS (version 26.0). A two-sided *p*-value < 0.05 will be considered statistically significant for primary analysis.

### 2.9 Safety monitoring and adverse event management

All AEs will be monitored, recorded, and graded for severity (CTCAE v5.0). Expected AEs include transient GI symptoms (diarrhea, bloating), procedure-related discomfort (sore throat, abdominal pain), and sedation risks. Management is primarily supportive. Any Grade ≥3 AE, SAE, or unexpected event potentially related to FMT must be reported to the PI and Ethics Committee within 24 hours. The study may be paused for safety review if a serious FMT-related infectious event occurs.

### 2.10 Quality control

Strict quality control is maintained for FMT preparation, including pathogen screening, viability testing (target ≥5 × 10^8^ viable cells/mL, ≥80% viability), and batch consistency monitoring. Standardized operating procedures govern all laboratory and clinical processes.

(1) Routine Stool Examination: Random samples are tested for pathogens and cell counts.
(2) Pathogen Detection: Negative results required for bacterial, parasitic, and viral pathogens; multidrug-resistant genes (e.g., ESBL, carbapenemase) must be absent.
(3) Viability Standards: ≥80% bacterial viability; ≥5 × 10^8^ viable cells/mL.
(4) Microbial Consistency:
  Uniform microbial composition within the same donor and batch.
  No statistically significant differences in microbiota between batches from the same donor within 6 months.
(5) Labeling: Each bottle must be independently packaged and labeled.

## 3 Discussion

This protocol describes a rigorous, triple-blind, randomized controlled trial designed to directly compare the efficacy and safety of two invasive FMT delivery routes - NJT and colonoscopy—in children with moderate-to-severe ASD. By employing a double-dummy design with sham procedures, the study aims to provide high-level evidence while controlling for placebo effects associated with invasive interventions, a notable challenge in previous FMT research.

The strengths of this study include its randomized, placebo-controlled design, the use of a single donor to ensure microbial homogeneity, comprehensive and blinded outcome assessments using CARS-2, and long-term follow-up to evaluate durability of effects and safety. The integrated metagenomic analysis will offer mechanistic insights by correlating microbial engraftment patterns with clinical response, potentially identifying predictors of success for each route.

A key challenge is maintaining blinding for participants undergoing different invasive procedures, although sedation and the double-dummy design mitigate this. The requirement for multiple sedated procedures also raises considerations regarding feasibility and participant burden. The sample size, while large, is justified to detect a clinically meaningful difference between three groups.

If successful, this trial will provide crucial evidence to guide the standardization of FMT protocols for ASD, informing the selection of the most effective and tolerable administration route. The findings will significantly advance the field of microbiota-based therapies for neurodevelopmental disorders and contribute to the development of future treatment guidelines.

A key methodological innovation in this trial is the modified colonoscopic FMT protocol. Instead of subjecting participants to three separate colonoscopies under anesthesia, this group undergoes a single colonoscopy for the initial microbiota infusion and the placement of a secured TET. The subsequent two treatments are delivered via this TET. This approach significantly reduces the procedural burden, anesthesia exposure, and associated risks for participants in the FMT-C arm, enhancing the feasibility and ethical profile of the study. Furthermore, it ensures that all infusions are delivered directly to the same target site (the cecum), which may promote more consistent microbial engraftment compared to protocols relying on oral capsules that must transit the harsh acidic environment of the stomach. We will closely monitor for potential complications related to the indwelling TET, such as discomfort, accidental displacement, or local irritation. The strength of the current study lies in its rigorous randomized controlled design, which directly addresses the key questions regarding placebo effects and route optimization raised by our previous single-arm study (Lin et al., 2025). By building upon that foundational work and introducing a placebo control group alongside two distinct active FMT routes (NJT vs. colonoscopy with TET), this study aims to provide higher-level evidence to determine the optimal FMT treatment strategy, thereby translating preliminary observations into actionable clinical guidelines.

Although we use a single primary donor to ensure homogeneity, donor switching during the trial, if necessary, could introduce biological variability. We plan sensitivity analyses to explore this potential confounder.

## Data Availability

The original contributions presented in the study are included in the article/supplementary material, further inquiries can be directed to the corresponding author.
